# Incidentally Detected Low-Grade Appendiceal Mucinous Neoplasm Following Laparoscopic Appendectomy for Acute Appendicitis

**DOI:** 10.7759/cureus.107008

**Published:** 2026-04-14

**Authors:** Oscar A Vazquez, Felipe Alberto Camacho Cordovez, Mridul Pansari, Jay Oberoi, Anupam Gupta

**Affiliations:** 1 Surgery, University of Florida, Gainesville, USA; 2 Pathology, Jackson Memorial Hospital, Miami, USA; 3 Hepato-Pancreatico-Biliary (HPB) Surgery, Virtua Medical Group, Dover, USA; 4 Internal Medicine, Jackson Memorial Hospital, Doral, USA; 5 Surgery, Bengaluru Medical College, Bengaluru, IND; 6 Surgery, Kempegowda Institute of Medical Science, Bengaluru, IND

**Keywords:** acute appendicitis, gastrointestinal oncology, histopathology, incidental finding, lamn, laparoscopic appendectomy, low-grade appendiceal mucinous neoplasm, pseudomyxoma peritonei, ptis

## Abstract

Appendiceal mucinous neoplasms are rare entities that account for a small proportion of all gastrointestinal malignancies and are most often discovered incidentally on histopathological examination of appendectomy specimens. We present the case of a 36-year-old woman who presented to the emergency department with acute right lower quadrant abdominal pain and was found to have acute appendicitis on computed tomography imaging. She underwent an uncomplicated laparoscopic appendectomy, and final pathology revealed a low-grade appendiceal mucinous neoplasm (LAMN) confined to the appendix, staged as pTis(LAMN). Postoperative colonoscopy was performed to evaluate the appendiceal orifice and exclude synchronous colorectal lesions. This case highlights the importance of routine histopathological examination of all appendectomy specimens and the need for appropriate postoperative surveillance in patients with incidentally discovered appendiceal mucinous neoplasms.

## Introduction

Acute appendicitis is one of the most common causes of abdominal pain presenting to the emergency department, with an annual incidence of approximately 100 per 100,000 individuals in Western countries and a lifetime risk of 6.7% to 8.7% [1,2]. Laparoscopic appendectomy remains the standard of care for the management of acute appendicitis. In the vast majority of cases, appendiceal inflammation results from luminal obstruction by fecaliths, lymphoid hyperplasia, or other benign causes. However, in a small subset of patients, the underlying etiology of appendicitis is an occult neoplasm [3].

Incidental appendiceal neoplasms are identified in approximately 1% to 2% of appendectomy specimens performed for acute appendicitis [3,4]. Among these, the histologic spectrum includes neuroendocrine tumors, low-grade appendiceal mucinous neoplasms (LAMNs), adenocarcinomas, and goblet cell adenocarcinomas [5,6]. LAMNs are defined as mucin-producing epithelial neoplasms characterized by low-grade cytologic atypia, villous or flat architecture, and a pushing pattern of growth with potential for mural fibrosis and mucin accumulation. These tumors are of particular clinical interest because, despite their low-grade histologic appearance, they carry the potential to progress to pseudomyxoma peritonei (PMP), a condition characterized by the accumulation of mucinous deposits throughout the peritoneal cavity [7]. The classification and management of appendiceal mucinous neoplasms have undergone significant evolution in recent years, with the World Health Organization (WHO) and the American Joint Committee on Cancer (AJCC) establishing updated nomenclature and staging criteria [7,8].

We present a case of a LAMN incidentally discovered on pathological examination following laparoscopic appendectomy for acute appendicitis in a young woman. This report discusses the current classification, staging, surgical management, and surveillance recommendations for this rare entity.

## Case presentation

A 36-year-old woman of African American origin with a body mass index of 35.45 kg/m² presented to the emergency department with a one-day history of acute abdominal pain associated with nausea and vomiting. Her past medical history was unremarkable for malignancies or colorectal disorders. Surgical history was notable only for a prior cholecystectomy. Family history was noncontributory.

**Figure 1:**
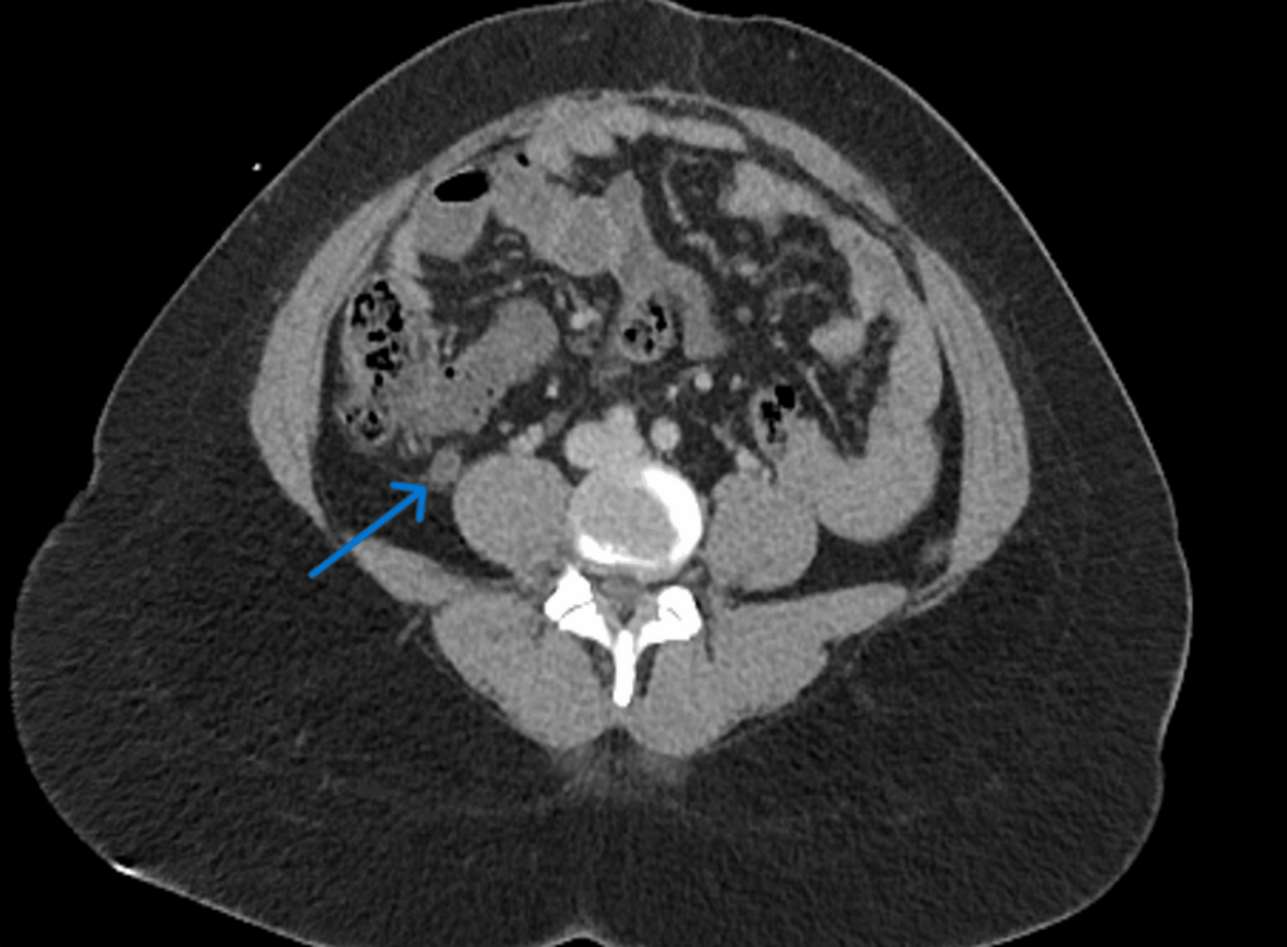
Axial computed tomography (CT) of the abdomen and pelvis demonstrating acute appendicitis. Axial CT image showing a dilated appendix (blue arrow) with surrounding periappendiceal fat stranding, consistent with acute appendicitis.

On examination, the pain was localized to the right lower quadrant. Clinical examination revealed right lower quadrant tenderness without peritoneal signs. Laboratory workup, including a complete blood count and comprehensive metabolic panel, was within normal limits. Given the clinical presentation, a computed tomography (CT) scan of the abdomen and pelvis was obtained, which revealed an inflamed and dilated appendix measuring 1.1 cm in diameter with periappendiceal fat stranding, consistent with acute appendicitis (Figure 1).

The patient was initiated on broad-spectrum intravenous antibiotics, and informed consent was obtained for a laparoscopic appendectomy. The procedure was performed using a standard three-port technique. Intraoperative findings demonstrated an inflamed and dilated appendix without evidence of perforation, abscess, mucin deposits, or other concerning findings within the abdominal cavity. The patient had an uneventful postoperative recovery and was discharged home on the first postoperative day.

**Figure 2:**
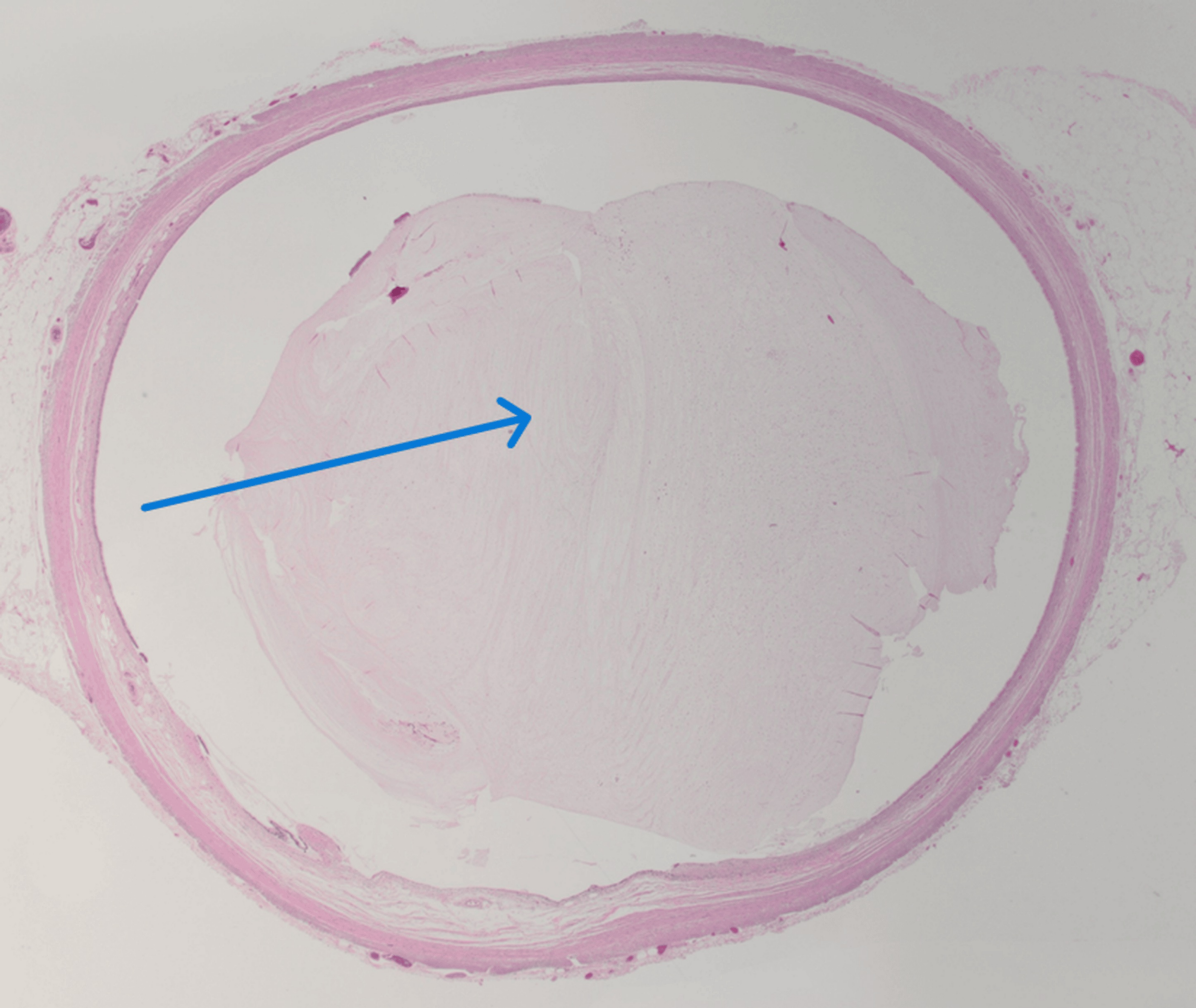
Histopathological examination of the appendix (hematoxylin and eosin stain, 1.25×). Low-power view demonstrating a markedly dilated appendiceal lumen filled with abundant extracellular mucin (blue arrow), consistent with a mucinous neoplasm.

Final pathological examination of the appendectomy specimen revealed a low-grade appendiceal mucinous neoplasm involving the distal half of the appendix. Histologic sections demonstrated a dilated appendiceal lumen filled with abundant mucin (Figure 2).

**Figure 3:**
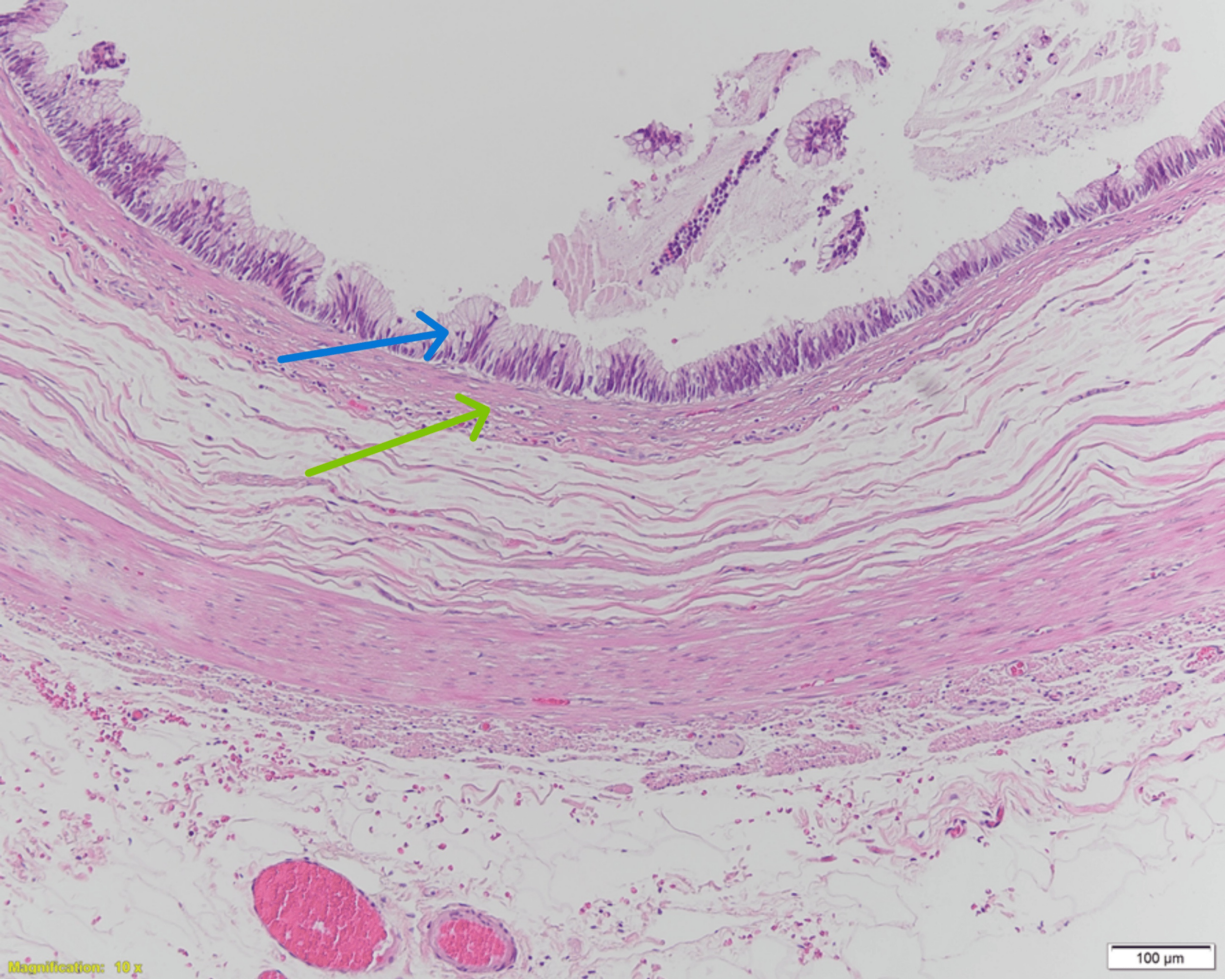
Histopathological examination of the appendix (hematoxylin and eosin stain, 10×). Intermediate-power view demonstrating mucinous neoplastic epithelium (blue arrow) circumferentially approaching the muscularis mucosae with associated obliteration of the lamina propria (green arrow), consistent with a low-grade appendiceal mucinous neoplasm.

The neoplastic mucinous epithelium circumferentially approached the muscularis mucosae with obliteration of the lamina propria (Figure 3).

**Figure 4:**
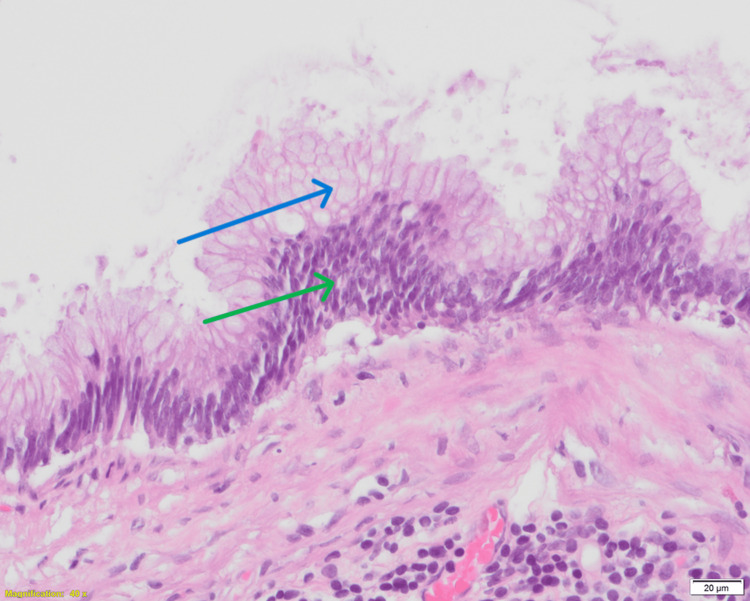
Histopathological examination of the appendix (hematoxylin and eosin stain, 40×). High-power view demonstrating mucinous epithelium with low-grade cytologic features, including abundant cytoplasmic mucin vacuoles (blue arrow) and nuclear pseudostratification (green arrow), consistent with a low-grade appendiceal mucinous neoplasm.

High-power examination showed low-grade cytologic features with cytoplasmic mucin vacuoles and nuclear pseudostratification (Figure 4).

The tumor was confined to the muscularis propria and was staged as pTis(LAMN) according to the AJCC staging system (Version 9) [9]. Surgical margins were negative for neoplasm.

**Figure 5:**
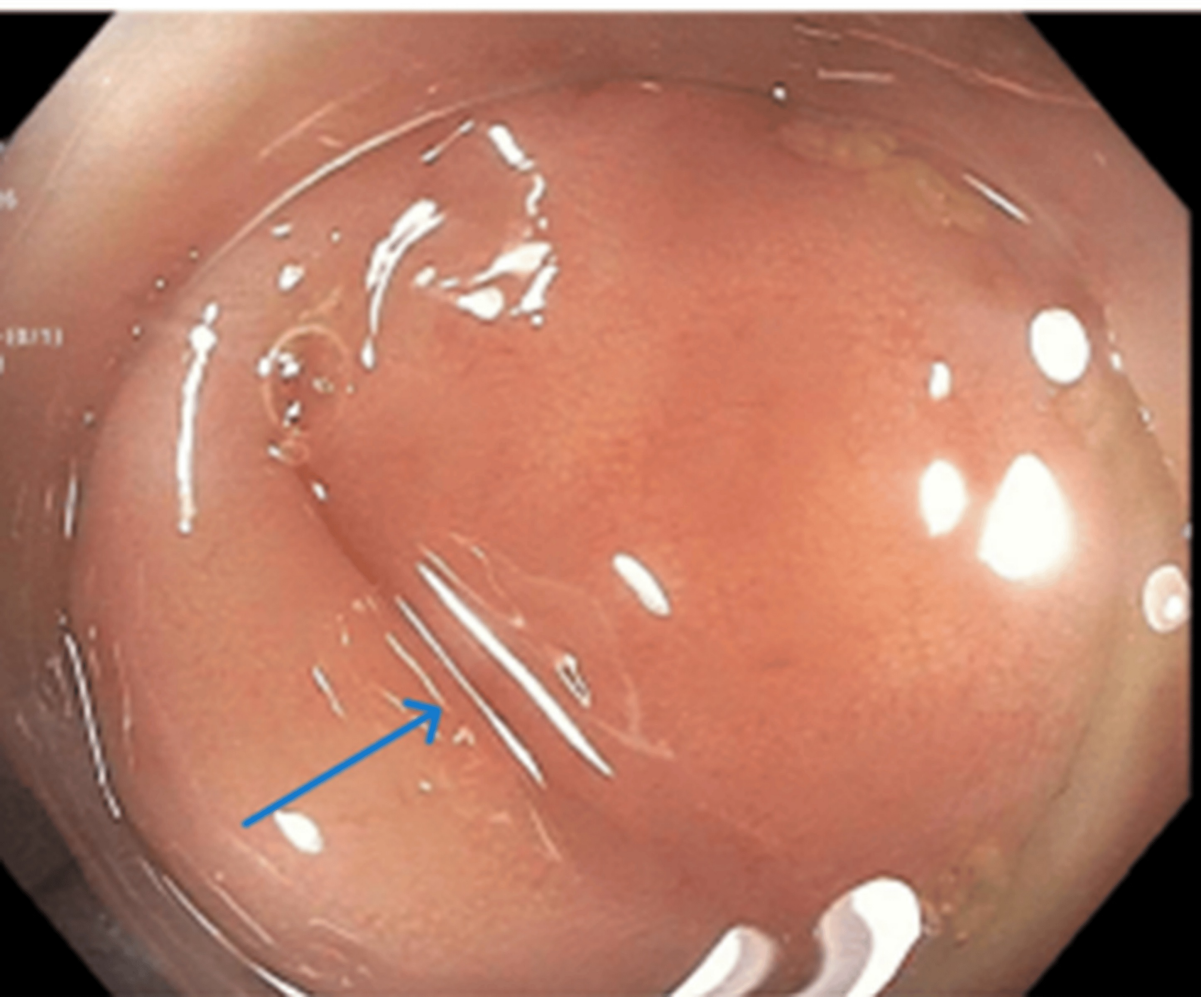
Colonoscopic view of the appendiceal orifice following appendectomy. Endoscopic image demonstrating a normal-appearing appendiceal orifice with no evidence of residual lesion or mucosal abnormality.

Following the pathologic diagnosis, the patient underwent a postoperative colonoscopy to evaluate the appendiceal orifice and exclude synchronous colorectal neoplasms, as recommended by current guidelines [5]. The colonoscopy demonstrated a normal-appearing appendiceal orifice (Figure 5) and no synchronous lesions.

Given the completely resected, localized LAMN (pTis) with negative margins and no high-risk features, the multidisciplinary tumor board recommended no routine surveillance imaging. The patient was advised clinical follow-up, with consideration of interval imaging only if symptoms develop. Notably, there were no preoperative clinical or radiologic features suggestive of an underlying neoplasm in this case. The diagnosis of low-grade appendiceal mucinous neoplasm (LAMN) was made incidentally on histopathological examination, highlighting the limitation of current diagnostic modalities in distinguishing LAMN from routine acute appendicitis.

## Discussion

Appendiceal mucinous neoplasms represent a rare but clinically significant subset of gastrointestinal tumors. Primary appendiceal neoplasms are most often found incidentally during appendectomy following clinical presentation of acute appendicitis [5,6]. The reported incidence of incidental appendiceal neoplasms in appendectomy specimens ranges from 1% to 2% in large series, with rates increasing with patient age [2-5]. In a recent multicenter registry study, incidental neoplasms were identified in 1.3% of appendectomies performed for acute appendicitis, with the rate rising from 0.6% in patients aged 18 to 39 years to 4.8% in patients 70 years and older [3-5]. A large single-center retrospective study similarly reported incidental appendiceal tumors in 1.6% of 2,293 appendectomy specimens, with neuroendocrine tumors being the most frequent histologic subtype, followed by LAMNs [2,3].

The classification of appendiceal mucinous neoplasms has been a subject of considerable debate. According to the most recent WHO classification, appendiceal epithelial neoplasms are categorized into serrated lesions and polyps, mucinous neoplasms, adenocarcinomas, goblet cell adenocarcinomas, and neuroendocrine neoplasms [4]. Current terminology for mucinous neoplasms includes LAMN, high-grade appendiceal mucinous neoplasm (HAMN), and mucinous carcinoma peritonei of varying grades [5]. Older terminology, such as mucinous cystadenoma, mucinous cystadenocarcinoma, and mucinous tumor of uncertain malignant potential, is no longer recommended [4-6].

Histologically, LAMNs are characterized by tall mucinous epithelial cells arranged in villous or flat configurations that invade with a distinct pushing border, in contrast to the infiltrative glands with desmoplasia seen in adenocarcinomas [5]. LAMNs are typically associated with atrophy of the appendiceal wall, loss of lymphoid tissue, and effacement of the muscularis mucosae [5-8]. Mucin pools are common and may extend into the serosa and beyond. Molecularly, LAMNs are almost always microsatellite stable, BRAF V600E negative, and frequently harbor KRAS mutations [5,9].

The staging of LAMNs follows unique conventions within the AJCC staging system (Version 9) [9]. LAMNs confined to the muscularis propria, including acellular mucin or mucinous epithelium, are staged as pTis(LAMN). Notably, pT1 and pT2 categories are not applicable to LAMN. Acellular mucin or mucinous epithelium extending into the subserosa or serosa is classified as pT3 or pT4a, respectively [9]. In the present case, the tumor was confined to the muscularis propria and was appropriately staged as pTis(LAMN).

The surgical management of localized LAMN is well established. According to the National Comprehensive Cancer Network (NCCN) guidelines, negative-margin appendectomy is largely considered sufficient for LAMN and HAMN [5]. Ileocecectomy may be considered when appendectomy or extended appendectomy is not feasible. Right hemicolectomy is reserved for histologies at higher risk of hematogenous spread, such as adenocarcinoma [5,8,10]. In the present case, the laparoscopic appendectomy achieved negative margins, and no further surgical intervention was required. The NCCN guidelines also note that there is no clear role for routine second-look laparoscopy in the absence of other suggestive risk factors for recurrent disease [5,9].

The prognosis of completely resected, localized LAMNs is excellent. In a large study of 337 patients with appendiceal mucinous neoplasms confined to the appendix, no disease recurrence, peritoneal involvement, or disease-specific mortality was observed after a median follow-up of 56.1 months, including cases with high-grade dysplasia [11]. Similarly, a single-institution study of 114 patients with localized LAMNs reported no tumor recurrence after a mean follow-up of 4.7 years, regardless of T-category or margin status [12]. A Mayo Clinic study of 125 patients with LAMNs confined to the appendix reported an overall recurrence rate of 4%, with 5- and 10-year cumulative recurrence incidence rates of 3% and 6%, respectively [13]. All recurrences in that study were PMP, and risk factors for recurrence included acellular mucin limited to the right lower quadrant and tumor size less than 2 cm [13].

Despite the favorable prognosis, the potential for progression to PMP warrants consideration. PMP involves the gradual accumulation of gelatinous peritoneal metastases, typically resulting from rupture of a mucinous appendiceal neoplasm, and is treated with cytoreductive surgery and hyperthermic intraperitoneal chemotherapy [5,8]. The NCCN guidelines note that for completely resected LAMN confined to the appendix without high-risk features such as extra-appendiceal cellular mucin or the presence of signet ring cells, surveillance may not be needed [5,6].

Colonoscopy is recommended following the diagnosis of an appendiceal neoplasm to rule out synchronous colorectal lesions [5,6]. A retrospective cohort study found that colorectal neoplasms were identified in a notable proportion of patients with mucinous and malignant appendiceal neoplasms during follow-up, underscoring the importance of colonoscopic evaluation [14].

This case illustrates several important clinical points. First, the preoperative diagnosis of appendiceal mucinous neoplasms remains challenging, as clinical and radiologic features are often indistinguishable from those of routine acute appendicitis. The CT findings in our patient demonstrated only a dilated, inflamed appendix without features suggestive of a mucocele or neoplasm. Second, the case reinforces the critical importance of routine histopathological examination of all appendectomy specimens, as the incidental discovery of a LAMN fundamentally alters the patient's follow-up trajectory. Third, the growing trend toward nonoperative management of uncomplicated appendicitis raises concerns about missed appendiceal neoplasms. A recent study found that clinically significant lesions requiring a change in management were identified in 0.31% of the total appendectomy cohort and 0.13% of patients eligible for antibiotic-only treatment [1].

## Conclusions

This case demonstrates the incidental discovery of a low-grade appendiceal mucinous neoplasm in a young woman undergoing laparoscopic appendectomy for acute appendicitis. The prognosis for completely resected, localized LAMN is excellent, and negative-margin appendectomy is considered adequate surgical treatment. Postoperative colonoscopy should be performed to exclude synchronous colorectal lesions. Clinicians should maintain a high index of suspicion for occult appendiceal neoplasms, particularly as nonoperative management of appendicitis becomes more prevalent, and should ensure that all appendectomy specimens undergo thorough histopathological evaluation.
